# Brain activity characteristics of RGB stimulus: an EEG study

**DOI:** 10.1038/s41598-023-46450-z

**Published:** 2023-11-03

**Authors:** Alireza Khadir, Mohammad Maghareh, Shamim Sasani Ghamsari, Borhan Beigzadeh

**Affiliations:** Biomechatronics and Cognitive Engineering Research Lab, School of Mechanical Engineering, Iran University of Science and Technology, Tehran, Iran

**Keywords:** Neuroscience, Cognitive neuroscience, Perception

## Abstract

The perception of color is a fundamental cognitive feature of our psychological experience, with an essential role in many aspects of human behavior. Several studies used magnetoencephalography, functional magnetic resonance imaging, and electroencephalography (EEG) approaches to investigate color perception. Their methods includes the event-related potential and spectral power activity of different color spaces, such as Derrington-Krauskopf-Lennie and red-green-blue (RGB), in addition to exploring the psychological and emotional effects of colors. However, we found insufficient studies in RGB space that considered combining all aspects of EEG signals. Thus, in the present study, focusing on RGB stimuli and using a data-driven approach, we investigated significant differences in the perception of colors. Our findings show that beta oscillation of green compared to red and blue colors occurs in early sensory periods with a latency shifting in the occipital region. Furthermore, in the occipital region, the theta power of the blue color decreases noticeably compared to the other colors. Concurrently, in the prefrontal area, we observed an increase in phase consistency in response to the green color, while the blue color showed a decrease. Therefore, our results can be used to interpret the brain activity mechanism of color perception in RGB color space and to choose suitable colors for more efficient performance in cognitive activities.

## Introduction

Visual perception includes the sensation of many object properties such as orientation, contrast, disparity, movement, surface information, shape discrimination, surface depth, and surface segmentation. The perception of color is a fundamental cognitive feature of our psychological experience, with a key role in many aspects of human behavior^1^.

The visual information processing is implemented by three groups of retinal ganglion cells (magnocellular, parvocellular, and koniocellular)^2,3^. They have different cone inputs from the presented color stimuli. Magnocellular retinal ganglion cells (parasol cells) receive their input from long- (L) and middle- (M) wavelength-sensitive cones^4^. On the other hand, Parvocellular retinal ganglion cells, or midget cells, receive opposed L and M cone inputs and transmit red-green chromatic information to associated brain cortices. Accordingly, the received input of koniocellular retinal ganglion cells is from short-wavelength sensitive (S) cones, which allow them to transmit blue-yellow chromatic information^5,6^.

Although the sensitivity spectra of human retinal cones is usually performed by invasive approaches such as ex-vivo retinal unit recordings^7^ and psychophysical color matching^8^, the color perceptual response is usually studied noninvasively in humans using methods such as magnetoencephalography (MEG)^9^ and functional-magnetic-resonance-imaging (fMRI)^10^. The research on human^11^ and non-human^12^ primates showed that visual stimuli often induce gamma-band rhythmic activity (neuronal gamma-band is 30–100 Hz, and induced gamma-bands is around 40 Hz for low-gamma and 80 Hz for high-gamma)in the early visual cortex which can be dependent on specific feature values in the presented visual stimuli such as contrast and color^9^. There is diversely reported induced gamma activity by different colors^9^. Accordingly, many studies showed strong gamma-band oscillations induced by red stimuli. For example, in the study of V1 and V4 recordings in anesthetized macaque, Rols et al.^13^ first described higher gamma power for red-colored stimuli compared to green and yellow ones. At the same time, they were heteroluminant and presented on a gray background. Additionally, Brunet et al.^14^ used natural stimuli while presenting 65 grayscale and color images to two awake macaque monkeys during electrocorticography (ECoG) recordings over V1 and V4 areas and found that the colored orange images on a gray background induced the highest gamma power.

Furthermore, Stauch et al.^9^ recorded MEG signals from the visual cortex of 30 human subjects while presenting stimuli with equal luminance and cone contrast levels. The results showed that, in contrast to previous studies, there was no significant difference in gamma-band oscillations of red and green stimuli of equal L–M cone contrast. Moreover, there appeared to be a weak gamma response for the blue stimuli with contrast exclusively on the S-cone axis, showing a smaller Event-Related-Field (ERF). These results suggest that when colored stimuli produce similar V1 input strength, the resulting signals have no significant preference for colors.

Although it is widely accepted that electroencephalography (EEG) is a non-invasive and relatively inexpensive method for assessing neurophysiological and cognitive functions^15^, there is a limited number of studies that explore the potential of extracting information about color perception from EEG signals. Moreover, studies experimented with EEG signals to identify the brain’s responses to colored stimuli mostly used the power spectral density, PSD^16^, functional connectivity methods^17^, and also investigated the EEG effective connectivity network^18^. Other limited available literature investigating the possibility of extracting color perception information with EEG signals used mostly Event-Related-Potentials (ERP) analysis^19–24^. They suggest that amplitude or latency changes in ERPs are assumed to reflect the effects of color categories.

In addition, several approaches are available for investigating color categorization in the cortex and testing the relation between behaviorally observed color categories and continuous neural activity measures. So far, there have been reports of category effects in the temporal dynamics of color processing using EEG^25–29^. Some EEG studies focused on the ERP approach, and the EEG signal was low-pass filtered under 40 Hz^30–34^. Furthermore, they used color stimuli that were mostly in green-blue boundaries of Munsell color system^35^, or CIELAB^36^ and CIELUV^37^ color space. Additionally, amplitude or latency changes in ERPs are assumed to reflect the effects of color categories^38^. For instance, early components affected by color categories, including the first positive (P1) and negative (N1) component and the visual mismatch negativity (vMMN), are assumed to be the result of perceptual processing stages^39^. Accordingly, later components are associated with post-perceptual, higher-level cognition, including the second and third positive (P2, P3) and negative deflections (N2, N3). Nevertheless, the assumption about purely perceptual modulations of early components (P1, N1, and vMMN) is now debated because it has been reported that spatial attention may enhance early ERP components such as P1 and N1 amplitudes^38^.

In recent decades, different aspects of the psychological effect of colors are also investigated^40–43^, for example, emotional effects^44–51^, colored multimedia learning^18,52^, cognitive performances^53^, and memory facilitation. Some authors reported that colored (red and blue) materials increased cognitive effort and caused positive emotion relative to achromatic materials^54^. Moreover, neutral colors such as green are reported to boost calmness and moderate, ordinary feelings, and warm colors in the red color system arouse warm, positive, active feelings^16^. Saturated and bright colors were reported to cause considerably stronger skin conductance responses, indicating higher arousal levels^55^. Furthermore, compared to colors with shorter wavelengths (like blue), colors with longer wavelengths (like red) evoked a higher level of arousal emotion^56–59^. Additionally, Yoto et al.^16^ used subjective emotional assessments and EEG and showed that red produces higher average power in theta and alpha rhythms in the frontal lobe. Ueda et al.^60^ found that blue stimulus inhibited the beta wave activity in the occipital areas, proposing the more relaxing effect of the blue.

While in daily life and common experineces, people deal with red-green-blue (RGB) color space, we found insufficient studies in RGB space. To the best of our knowledge, there is a lack of a comprehensive study investigating all aspects of RGB color’s EEG signals, such as power, phase, and oscillation components. Concerning the importance of revealing perceptual color information processing^47,61–64^ and fewer studies focusing on RGB space, we investigated the time-dependent mechanisms of color perception with EEG data.

## Materials and methods

The present study has been approved by the Iran University of science and technology and its ethics committee and was conducted in accordance with the declaration of Helsinki^65^. Meanwhile, before each test, subjects signed an informed consent explaining the whole scenario of the experiment in detail, in accordance with the local ethics committee. All the applied procedures conform to the Declaration of Helsinki (1964) of the World Medical Association concerning human experimentation.

### Participants and setup

In this study, twelve subjects (9 males, 3 females), all right-handed, participated voluntarily. Their ages ranged from 20 to 28 years, with a mean age of 24.4 years (SD = 2.5). Participants in this and all subsequent experiments were naive to the experimental paradigm, had no previous neurological and psychiatric disorders, and reported normal or corrected-to-normal color vision.

In this study, an a priori power analysis was performed using G*Power version 3.1.9^66^ to determine the appropriate sample size. The analysis aimed to ensure adequate statistical power (0.8) to detect a significant effect in a within-subject repeated measures (rm)ANOVA with three levels of condition, using a significance level $\alpha$ of 0.05. The power analysis considered a correlation among repeated measures $\rho$ of 0.5 and an effect size (Cohen’s d) of 0.4. As a result, a sample size of twelve subjects was determined to be sufficient to meet these criteria.

### Apparatus and stimuli

In this experiment, we used a cathode-ray tube (CRT) monitor with a resolution of 1024 $\times$ 768 pixels and a 60 Hz refresh rate. The monitor’s gamma and color calibration parameters were extracted using an X-Rite ColorMunki Display device (www.xrite.com) along with DisplayCAL software (www.displaycal.net); in particular, gamma correction values for RGB colors were cosidered to be $\gamma _R = 2.77$, $\gamma _G = 2.79$, and $\gamma _B = 2.76$, respectively. Moreover, RGB stimuli are isolominance with $L = 8.99$ cd/m$^2$. This results in the RGB values presented to participants as R = [181 0 0], G = [0 124 0], and B = [0 0 255]. The subjects sat on a comfortable chair in a fully dark room covered with black color and approximately 70 cm from the monitor. Before each session, the subject was asked to relax for 1 minute to achieve a calm situation.

**Figure 1:**
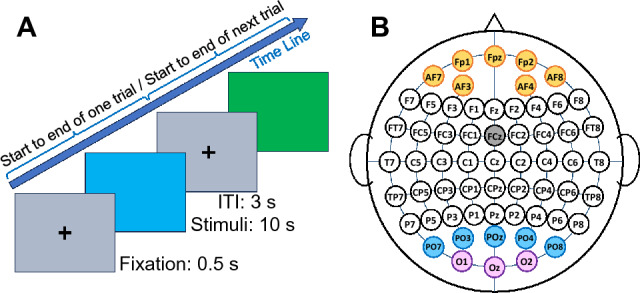
Visual paradigm and electrode position. (**A**) The visual paradigm consists of randomly selected stimuli, starting with a 0.5 s fixation point, after which participants were presented with a color (RGB) screen for 10 s followed by a 3 s delay period of gray background. (**B**) The location of the electrodes is based on the 10-20 system, and the areas marked on the electrodes are the regions mentioned in this paper. The occipital area includes O1, Oz, and O2 electrodes that are marked with the purple; the occi-parietal region includes O1, Oz, O2 (all marked with purple), and PO3, PO4, POz, PO7, PO8 (marked with blue); prefrontal area (AF7, AF3, AF4, AF8, Fp1, Fpz, Fp2) marked with orange.

We presented RGB colors randomly to each subject 5 times, each time for a duration of 10 seconds, aligned with previous related studies^67–69^. After each presentation, the screen briefly switched to a gray color for 3 seconds, displaying a white cross sign at its center. Following this interval, the next color was presented with the same timing, and this sequence was repeated a total of 5 times; see Fig. 1A.

### Electroencephalograph (EEG) aquisition

A 64-channel ANT neuro EEG signal acquisition device (ANT Neuro, Hengelo, The Netherlands) was used with a sampling rate of 512 Hz. The electrodes used here were monopolar Ag/AgCl electrodes placed on scalp with a cap according to the standard 10–20 system. Figure 1B shows the positions of the electrodes. Two electrodes (A1 and A2) were connetcted to mastoide and their avereage was considered as reference; the ground was connected to FCZ. The electrodes PO5 and PO6 were not used; finally, we employed the date gathered from remaining 59 channels.

### EEG preprocessing

The EEG pre-processing and processing was performed by custom-written Matlab scripts and commands from the EEGLAB toolbox^70^. We applied a highpass filtered at 1 Hz on EEG raw data by using “eegfilt” in the EEGLAB toolbox. First, raw data were imported into MATLAB 2018a using “pop_loadset()”. Then, to extract epochs, the EEG signals were segmented from 1000 ms before to 2000 ms after stimulus onset followed by re-referencing the data to the average of the mastoids. To remove artifacts from the data, we used FASTER (Fully Automated Statistical Thresholding for EEG artifact Rejection^71^) algorithm, which uses a statistical threshold for reject bad channels and epochs. To eliminate eye blinks and muscle activities, we first extracted the EEG components of each subject with the “pop_runica” EEGLAB command, then removed each of the noisy components with visual inspection of the spectrum and topoplot. It is notable that we remove at most one or two of the ICA components in each subject. The main tool for selecting bad components is manual visual inspection; along with that, two toolboxes of FASTER and ADJUST are used. But the final decision is based on manual visual inspection. After removing artifact, the raw signal is compared to the clean signal by visual inspection. If the removing of a component results in any changes near the onset (under 200 ms after onset), that removal is ignored.

### ERP, ERO and time-frequency analysis

The EEG signal was bandpass filtered to capture only frequency-specific activity, with “eegfilt()” from EEGLAB Toolbox, which uses an order of $3*fix(Fs/low\,cutoff)$ zero-phase FIR bandpass filter.

We chose to employ time-frequency analysis methods instead of the power spectral density (PSD) method. Time-frequency analysis methods offer a higher temporal resolution for detailed and localized spectral changes over time, frequency specificity to examine changes in specific frequency bands, and flexibility in parameter selection. We utilized the Hilbert transform and Morlet wavelet methods for localizing power and phase in both time and frequency domains.

The Hilbert transform extracts a complex signal from a signal that contains only a real part, and it can be represented using Euler’s formula: $M(t)e^{i2\pi ft}$. The $M(t)$ is extracted as the instantaneous amplitude, and the following syntax is used to extract the complex analytic signal:

1 $$\begin{aligned} {hilbert(eegfilt(data, Fs, low\,cutoff, high\,cutoff) ' )'} \end{aligned}$$

where $Fs$ is the sampling frequency (512 Hz), and we used delta (1−4 Hz), theta (4−8 Hz), alpha (8−12 Hz), beta (12−30 Hz), low gamma (30−50 Hz), and high gamma (70−150 Hz) for the lower and upper bound. We used the mean() of trials under 6 Hz for ERP analysis, and the mean() of trials for each filtered frequency band was computed for the Event-related-Oscillation (ERO) analysis of their activity. Inter-Trial Phase Clustering (ITPC) was used for the examination of cortical phase synchrony or for measuring the phase coherency over trials. ITPC measures the extent to which a distribution of phase angles at each time-frequency-electrode point across trials is nonuniformly distributed in polar space. The Mathematical representation of ITPC is as follows:

2 $$\begin{aligned} {ITPC_{tf} = n^{-1} \sum ^{n}_{r=1} e^{i K_{tfr}} } \end{aligned}$$

where $n$ is the number of trials, $e^{ik}$ is taken from Euler’s formula and provides the complex polar representation of a phase angle $k$ on trial $r$ at time-frequency point $tf$. The syntax for computing the $k$ is as follows:

3 $$\begin{aligned} {angle(hilbert(eegfilt((data, Fs, low\,cutoff, high\,curoff) '))} \end{aligned}$$

ITPC is bounded by 0 and 1, with 0 indicating random phases and 1 indicating perfect phase coherency. In addition, because of the Gaussian shape of the frequency response of Morlet wavelets, wavelet convolution tends to produce smooth-looking and, therefore, easily visually interpretable time-frequency plots (Cohen^72^). We performed a fine-grained complex Morlet wavelet convolution to the EEG epochs to analyze the data in the time-frequency domain with frequencies ranging from 2 to 80 Hz in 80 logarithmically spaced steps.

### Topographical maps

To illustrate the different ERO and time-frequency measures of the scalp distributions in each time window of interest, we used topographical maps (EEGLAB topoplot() procedure^70^ involving ”biharmonic” spline interpolation) for power and ITPC value differences between the colors.

### Statistical analyses

To compare the ERO for each pair of colors, we performed a two-sided Wilcoxon test for each time point, which does not make assumptions about the underlying distribution. Other alternatives include paired t-test; the Wilcoxon signed-rank test is preferred over the paired t-test in cases where the data violate assumptions of normality, and involve small sample sizes. It provides a robust and flexible alternative for comparing paired observations, particularly when parametric assumptions are not met. We implemented a whole-subject permutation method. The permutation tests provide a robust and flexible approach for analyzing ERP data. They accommodate the complex nature of ERP data, control for multiple comparisons, enable the investigation of temporal effects, and are suitable for small sample sizes. The nonparametric nature of permutation tests makes them well-suited for ERP analysis, where the distributional assumptions may not be met, and the focus is on capturing the temporal dynamics and characteristics of the measured brain responses^73^. The whole-subject permutation method is an optimized and faster version of the permutation test, in which the sign of the ERO of each subject is randomly inverted in each iteration^74^; for each time point, we obtain a distribution that can derive the P value of the null hypothesis.

For multiple comparison corrections over time, we have some alternatives including False Discovery Rate (FDR), Bonferroni, and cluster-based methods; we used a cluster-based permutation analysis^75^ because the cluster-based methods offer improved sensitivity, control over type I error, flexibility, and interpretability, making them a preferred choice for multiple comparison correction in ERP analysis^73,76^. As a result, we used cluster-based permutation analysis with 1,000 permutations at an evaluation threshold of 0.05 on the data. The above method was extended to compare ERO activity for multiple colors and applied a Non-parametric Friedman Test (Similar to the parametric repeated measures ANOVA). The same procedure was used for power, and ITPC values. To evaluate the statistical significance of the resulting time-frequency analysis, the above procedure was repeated for each time point and frequency.

Furthermore, to evaluate the statistical significance of Topographical maps, we implemented the whole-subject permutation analysis with a two-sided Wilcoxon test used for 59 electrodes with n = 5000 permutations and a cluster-forming significance threshold of *p* < 0.01.

### Data-driven approach

We used exploratory data-driven approaches to find the significant differences in the RGB color information. The ERO, power, and phase coherency (ITPC) values of each channel are extracted with the Hilbert transform method to compare the different RGB stimuli in 6 band frequencies (delta, theta, alpha, beta, low gamma, high gamma). To produce particular regions of interest, the resulting data of 59 electrodes from a given color were averaged (e.g., the mean value of O1, OZ, and O2 for the occipital area). This leads to the generation of Occipital, Parietal, Occi-parietal, Central, Centro-Parietal, Frontal, Centro-Frontal, Prefrontal, and Temporal. Next, we applied the Wilcoxon signed-rank test to each time point for each pair of stimuli. We inspected different brain areas and band frequencies for meaningful significance regarding ERO components, power, and ITPC. A set of follow-up analyses was conducted on the extracted p-values, with the correction for multiple comparisons.

The outcome of an exploratory analysis showed a noticeable and reliable effect in the power of the occipital theta-band. In addition, we observed two strong and efficient effects in the ITPC analysis of the prefrontal area in the range of delta and theta-band. In the next step, we focused on these three spatial-frequency regions and applied a Non-parametric Friedman Test to compare the color information. Remarkably, we found a significant difference between ERO data in beta frequency.

## Results

**Figure 2:**
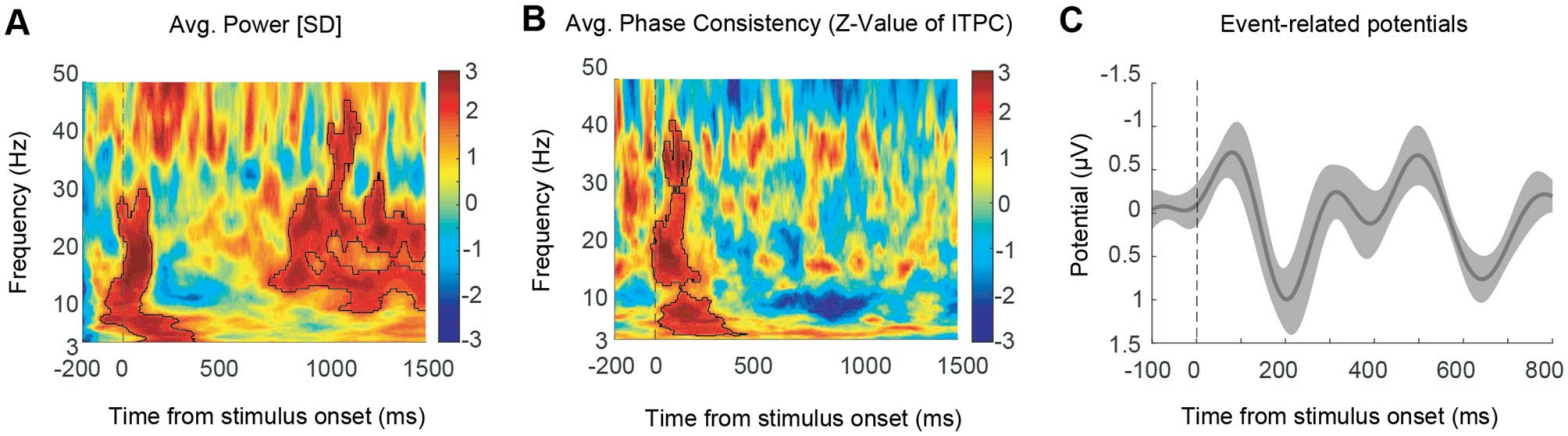
Neural EEG responses to visual stimuli in occipital (O1, Oz, and O2) electrodes. (**A**) Average time-frequency power plots of 12 participants around stimulus onset using the Morlet Wavelets Convolution method. Plots are averaged over all color conditions and based on z-scored baseline normalization (300–100 ms time-window before stimulus onset). In the map, red indicates that the power is higher than baseline periods, and blue indicates the opposite. Highlighted areas with the solid black outline indicate significant clusters under 30 Hz and shows a significant power increase immediately after stimuli onset (permutation test with 1000 permutations, participant = 12, cluster-forming threshold *p* < 0.05, corrected significance level *p* < 0.05). Dash lines indicate the time of stimulus onset (t = 0 ms). (**B**) Average time-frequency plots of z-scored p-values of ITPC for 12 subjects with the Morlet Wavelets Convolution method. Plots are averaged over all color conditions, and for each frequency, values are subtracted from baseline periods (300 - 100 ms time window before stimulus onset). The highlighted cluster showed a significant increase of under 40 Hz activity immediately after the presentation of the stimulus (permutation test with 1000 permutations, participant = 12, cluster-forming threshold *p* < 0.05, corrected significance level *p* < 0.05). (**C**) Event-related potentials for averaged over all color conditions (low-pass filter under 6 Hz).

Figure 2 illustrates the neural EEG responses to visual stimuli in occipital (O1, OZ, and O2) electrodes. The purpose of producing this plot is to demonstrate the response of the early visual sensory areas to the stimulus presentation. All color signals for each participant are integrated to extract the mean power, phase, and ERP of the data. In Fig. 2A, the average time-frequency power of 12 participants produced by the Morlet Wavelets Convolution method, and based on z-scored baseline normalization (300–100 ms time-window before stimulus onset) is illustrated. The plot shows a significant power increase under 30 Hz immediately after stimulus onset shown in Supplementary 2A and another significant cluster in the beta band (12–30 Hz) in late periods depicted in Supplementary 2B (permutation test with 1000 permutations, participant = 12, cluster-forming threshold *p* < 0.05, corrected significance level *p* < 0.05). The average time-frequency plots of z-scored p-values of ITPC for 12 subjects with the Morlet Wavelets Convolution method are demonstrated in Fig. 2B.

To calculate the z-scored values, first, the ITPC values are subtracted from baseline periods (300–100 ms time window before stimulus onset) for each frequency band. Then the non-parametric Wilcoxon test and within-subject analysis are performed, and the resulting z-values of each time-frequency point are shown in Fig. 2B. As depicted, the highlighted cluster exhibits a significant increase of under 40 Hz activity immediately after the stimulus presentation (permutation test with 1000 permutations, participant = 12, cluster-forming threshold p < 0.05, corrected significance level *p* < 0.05). Figure 2C presents the ERP of under 6 Hz, and the components of N75 ($\sim$70 ms) and P200 are evident.

**Figure 3:**
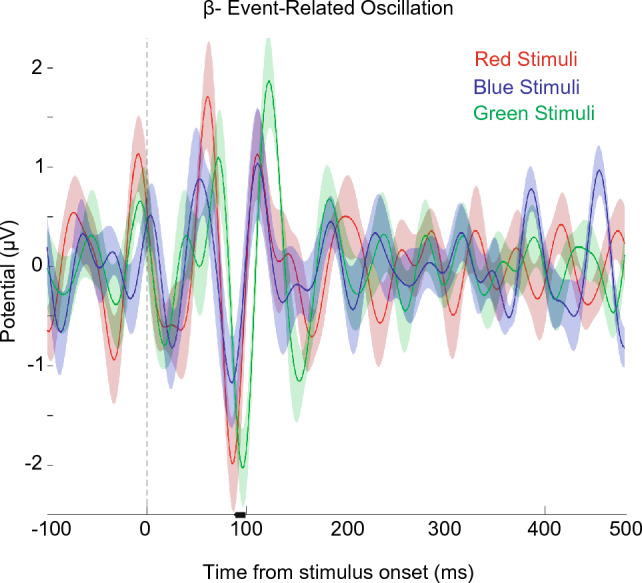
Event-related oscillation in the beta-band (12–30 Hz) in occipital (O1, Oz, and O2) electrodes. Each line shows beta ERO values over time. Dash lines indicate the time of stimuli onset with Error shadings showing $95\%$ confidence intervals, calculated across participants (n = 12). The dashed line indicates the time of stimuli onset. As depicted, the green-colored stimulus shows a latency shifting relative to blue and red stimuli approximately 100 ms after stimulus onset. The Horizontal black line shows the clusters of significant differences between RGB’s ERO in the time window of 88–98 ms (within-subject Friedman Test, 1000 permutations, participant = 12, cluster-forming threshold *p* < 0.01, corrected significance threshold *p* < 0.05).

To find the difference in the ERO of RGB colors, we first investigated the occipital oscillation of each 6 frequency band. The Supplementary 1 demonstrates the grand average ERO waveform in response to the RGB stimulus of each frequency band in occipital region. As depicted, only beta-band oscillation showed a meaningful difference with comparison-correction (permutation test with 1000 permutations, participant = 12, cluster-forming threshold p < 0.01, corrected significance threshold *p* < 0.05). For further analysis, the beta-ERO is illustrated in Fig. 3. There is an evident significant difference between the ERO of RGB colors, in which the trough of green color exhibits a latency shifting around 100 ms (88 to 98 ms) after the stimulus onset in contrast to other colors (a within-subject Friedman Test, cluster-based method for multiple comparison-correction, 1000 permutations, participant = 12, cluster-forming threshold *p* < 0.01, corrected significance threshold *p* < 0.05). This is a new discovery that was not mentioned in the previous RGB color analysis studies.

**Figure 4:**
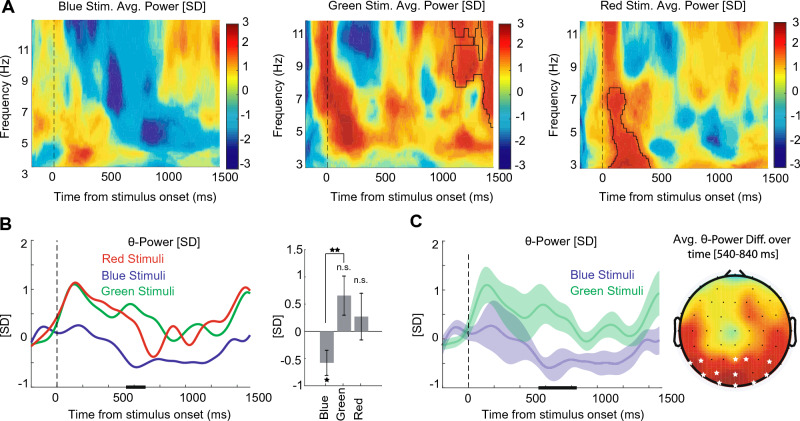
RGB power Information in theta-band frequency of the occipital (O1, Oz, and O2) electrodes. (**A**) Average time-frequency power plots of 12 participants with Morlet Wavelets Convolution and based on z-scored baseline normalization (300–100 ms time-window before stimulus onset) for blue(left), green(middle), and red(right). The plots are zoomed-in under 12 Hz (including delta, theta, and alpha bands) for more details. Highlighted areas with the solid black outline indicate significant clusters. (**B**) An average of power values from 12 participants in the theta band frequency (4–8 Hz). (Left) Each line shows the average of power values over time. The dashed line indicates the time of stimuli onset. The Horizontal black lines show the clusters of significant differences between RGBs in the 540 to 680 ms time window. (Right) Bar plot for averaged power values across the time window of significant periods in left side (540–680 ms) of each color. Error bars indicate $95\%$ confidence intervals calculated across participants (n = 12). The asterisks indicate significant differences in power value from zero (Wilcoxon rank sum test). Asterisks above horizontal lines indicate significant differences between each pair of colors (Post-hoc two-sided Wilcoxon tests). n.s : *p* > 0.05, *p < 0.05, **p < 0.01, ***p < 0.001. (**C**) the average power values for blue and green colors. (left) Same as panel **B** -left, with Error shadings showing $95\%$ confidence intervals, calculated across participants (n = 12). The Horizontal lines show clusters of significant differences. (Right) Topographies show the associated differences between the color blue vs. green in theta band in the 540–840 ms window. A star indicates O2-O1-Oz-POz-PO8-PO3-PO7-PO4-P7-P6-P5-P2 as occi-parietal significant sites.

In the power analysis, we found a distinguishable effect for the perception of colors in the occipital region, illustrated in Fig. 4. Figure 4A shows the average time-frequency power plots of 12 participants in the occipital (O1, Oz, and O2) electrodes with Morlet Wavelets Convolution and based on z-scored baseline normalization (300–100 ms time-window before stimulus onset) for blue(left), green(middle) and red(right), and for details, the plots are zoomed-in under 12 Hz. The plot demonstrates a significant power increase immediately after stimulus onset for red-colored stimulus, in addition to a significant cluster in the late period for green stimulus (permutation test with 1000 permutations, participant = 12, cluster-forming threshold *p* < 0.05, corrected significance level *p* < 0.05).

A power increase is also visible in the green and blue colors after the presentation of stimuli; however, the statistical analysis showed no significance. In the Time-Frequency plot, it is noteworthy that we observed a power suppression for blue color in the theta-band that is visible for approximately a long duration of time (0.4–1.4s); however, the statistical analysis of this particular region showed no significant difference. Nevertheless, this region of time-frequency had a meaningful difference in comparison between colors, which we further analyzed in panels B and C.

In Fig. 4B-left, each line shows the power average for each RGB color in theta-band frequency (4–8 Hz). We observed that the power value of the blue decreased remarkably relative to the baseline activity (300–100 ms time-window before stimulus onset) that holds for rather a long period, and this was not visible in red or green colors. With statistical analysis there is a significant differences between colors in the 540–680 ms time window (a within-subject Friedman Test, n = 1000 permutation, participants = 12, cluster-forming threshold *p* < 0.05, corrected significance threshold *p* < 0.05).

Figure 4B-right, depicts the bar plot of averaged power values across the time window of significant periods in panel B-left (540–680 ms) for each color: blue mean value $-\,0.55 \pm 0.2$, green mean value $0.62 \pm 0.34$, and red mean value $0.25 \pm 0.41$. A Friedman Test showed a significant difference between colors ($\chi ^2(2) = 6$, *p* < 0.05). Post-hoc two-sided Wilcoxon tests showed a statistically significant difference between the blue and green (Z = 2.67, *p* < 0.008). There was no significant effect on other paired colors.

Figure 4C-left demonstrates only the power values of blue and green colors with the most significant difference in Panel B. We illustrated the meaningful differences in a time window of 540–840 ms (two-sided Wilcoxon tests, 1000 permutations, cluster-forming threshold Z > 2.58, the corrected significance threshold *p* < 0.05). Figure 4C-right shows Z-valued topographies of the two-sided Wilcoxon test for the blue and green color in theta-band power at 540–840 ms after the stimulus onset. The statistical analysis indicates that occi-parietal electrodes (O2-O1-Oz-POz-PO8-PO3-PO7-PO4-P7-P6-P5-P2) are significant site (5000 permutations, cluster-forming threshold Z > 2.28, corrected significance threshold *p* < 0.01).

**Figure 5:**
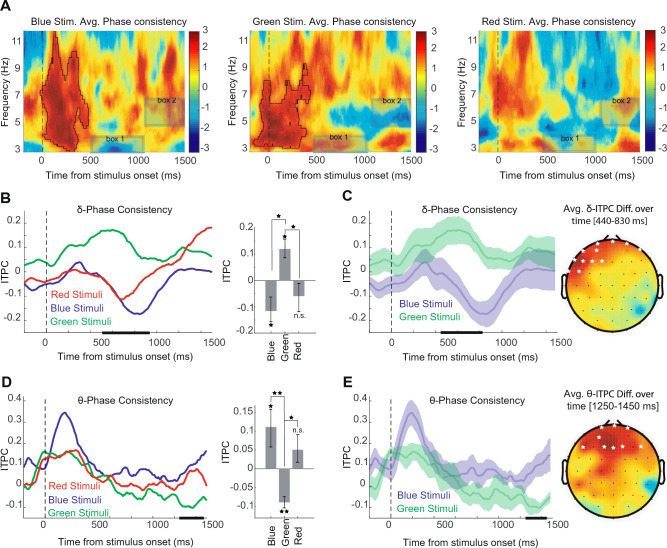
RGB phase information in delta & theta-band frequency in prefrontal (AF7, AF3, AF4, AF8, Fp1, Fpz, Fp2) electrodes. (**A**) Average z-scored p-values for ITPC for 12 subjects for blue(left), green(middle), and red(right). Highlighted areas with the solid black outline indicate significant clusters. (**B**) An average of ITPC values from 12 participants in the delta band frequency (2–4 Hz). (Left) ITPC values over time. The dashed line indicates the time of stimuli onset. The Horizontal black lines show the clusters of significant differences between RGBs in the time window of 500–940 ms. (Right) Bar plot for averaged ITPC values across the time window of significant periods in left side (500–940 ms) of each color. Error bars indicate $95\%$ confidence intervals calculated across participants (n = 12). The asterisks indicate significant differences in ITPC value from zero (Wilcoxon rank sum test). Asterisks above horizontal lines indicate significant differences between each pair of colors (Post-hoc two-sided Wilcoxon tests). n.s : *p* > .05, *p < 0.05, **p < 0.01, ***p < 0.001. (**C**) The average ITPC values for the colors blue vs. green with Error shadings showing $95\%$ confidence intervals, calculated across participants (n = 12). The Horizontal lines show clusters of significant differences. (Right) Topographies show the differences between the blue and green in the delta band in the 440–830 ms window. The star indicates prefrontal are significant sites. (**D**) An average of ITPC values for theta band frequency (4–8 Hz). (Left) ITPC values over time. The Horizontal black lines show the clusters of significant differences between RGBs in the time window of 1230–1470 ms. (Right) Bar plot for averaged ITPC values across the time window of significant periods in left side. (**E**). The average of ITPC values for the colors blue and green with Error shadings showing $95\%$ confidence intervals, calculated across participants (n = 12). The Horizontal lines show clusters of significant differences. (Right) Topographies show the associated differences of the color blue vs. green in theta band in the (1250–1450 ms) window. The star indicates a greater prefrontal activity.

We also performed the phase consistency analysis in the prefrontal area and observed a reliable outcome in the perception of RGB colors (Fig.5). Figure 5A demonstrates the average time-frequency plots (blue(left), green(middle), and red(right)) of ITPC p-values that are z-scored for 12 subjects, and we used Morlet Wavelets Convolution method. For each frequency, values are subtracted from baseline periods (300–100 ms before stimulus onset), and for details, the plots are zoomed-in under 12 Hz. The highlighted cluster exhibits a significant increase of under 12 Hz activity for blue and under 8 Hz for green immediately after the stimulus presentation (permutation test with 1000 permutations, participant= 12, cluster-forming threshold p < 0.05, corrected significance level *p* < 0.05). Although we also observed an increase in the phase consistency of the red color (under 12 Hz, shown in Fig. 5A-right), it was not meaningful after statistical analysis. We must mention that this initial phase coherency was not meaningful in comparison between colors. Moreover, it is also evident that around 600–1000 ms after stimulus onset, in low-frequency (under 4 Hz), the green color shows an increase of ITPC relative to the baseline activity. Interestingly, these results are opposite in red and blue colors, with negative ITPC values relative to the baseline duration (boxed region No.1 in Fig. 5A). To investigate this effect, we performed a statistical analysis of the delta-band for all three colors that are visible in panels B and C. Furthermore, in the late periods after onset (1100- 1500 ms) in the 4-8 Hz, it is visible that the green ITPC value decreases relative to the baseline activity, while red and blue have positive values (see Fig. 5A, box No.2). The result of statistical analysis of this effect is further investigated and illustrated in the panel D and E.

In Fig. 5B-left, each line shows the ITPC average for each RGB color in delta-band frequency (2–4 Hz). We observed that the red and blue-colored stimuli exhibit the same behavior in the first 500 ms post-stimulus onset, and have negative peak values relative to baseline (600–800 ms after onset). However, this trough happens later for the blue than red, but with a larger amplitude. On the other hand, the green showed an opposite behavior in ITPC values, in contrast to red and blue, and had a positive peak approximately 400–800 ms after onset. Additionally, statistical analysis showed a significant difference between colors in the 500 to 940 ms (a within-subject Friedman Test, 1000 permutations, participants =12, cluster-forming threshold *p* < 0.05, corrected significance threshold *p* < 0.05).

Figure 5B-right depicts the bar plot of averaged ITPC values across the time window of significant periods in panel B-left side (500–940 ms). The values of each color are as follows: blue mean value $-\,0.12 \pm 0.05$, green mean $0.12 \pm 0.04$, and red mean $-\,0.06 \pm 0.05$. A Friedman Test showed a significant difference between colors ($\chi ^2(2)=6.5$, *p* < 0.04). Post-hoc two-sided Wilcoxon tests showed a statistically significant difference between the blue and green (Z = 2.35, *p* < 0.02) and green vs. red (Z = 2.19, *p* < 0.03). However, there was no evident effect in red and blue.

Figure 5C-left shows the ITPC values of blue and green colors. We observed significant differences in a time window of 440–830 ms (two-sided Wilcoxon tests, 1000 permutations, cluster-forming threshold Z > 1.96, the corrected significance threshold *p* < 0.05). In Fig. 5C-right, the topoplots of Z-value for two-sided Wilcoxon of the color blue and green in delta-band power is illustrated (at 440–830 ms after the stimulus onset). The prefrontal area (F7-F5-Fp1-FT7-Fp2-Fpz-FC5-AF7-AF3-AF8-F3-F1) are significant site (5000 permutations, cluster-forming threshold Z > 1.96, corrected significance threshold *p* < 0.05).

In Fig. 5D-left, we demonstrated the ITPC average in theta-band frequency (4–8 Hz). Notably, after 200 ms post-stimulus onset, all colors showed similar behavior. Still, after reaching 1000 ms, the green gradually exhibits negative ITPC values (relative to baseline) (see Fig. 5A- middle panel and box 2). Within-subject Friedman Test analysis showed significant differences between colors in the time window of 1230 to 1470 ms (permutation test with n = 1000, cluster-forming threshold *p* < 0.05, corrected significance threshold p < 0.05).

The bar plot of averaged ITPC values across the time window of significant periods in panel D-left side (1230–1470 ms) is illustrated in Fig. 5D-right. The values of each color are as follows: blue mean value $0.11 \pm 0.05$, green mean $-\,0.09 \pm 0.03$, and red mean $0.05 \pm 0.04$. The Friedman Test showed a significant difference between colors ($\chi ^2(2)=9.5$, *p* < 0.009). The Post-hoc two-sided Wilcoxon tests showed a statistically significant difference between the blue vs. green (Z = 2.82, *p* < 0.005) and green vs. red (Z = 1.96, *p* < 0.05). However, there was no significant effect in red vs. blue.

Figure 5E-left shows the ITPC values of blue and green colors. The result demonstrates significant differences in a late period in the time window of 1250- to 1450-ms (two-sided Wilcoxon tests, 1000 permutations, cluster-forming threshold Z > 1.96, the corrected significance threshold *p* < 0.05). Furthermore, the Z-valued topoplots of two-sided Wilcoxon for blue and green color is shown in Fig. 5E-right, which is the theta-band power during 1250-1450-ms after the stimulus onset. The prefrontal area (F1-Fz-F5-Fp2-AF3-F4-Fp1-F2-Fpz) are significant sites (5000 permutations, cluster-forming threshold Z > 2.27, corrected significance threshold *p* < 0.01).

## Discussion

In our daily lives, we are constantly exposed to a rich variety of RGB colors, each with potential implications for brain processes. However, comprehensive investigations that consider factors such as response speed, power, and phase synchrony, and explore the effects of RGB colors across different brain regions, are notably limited in the current body of research. In this study, we harnessed EEG data to individually examine the influence of RGB colors on the human brain. Our findings reveal novel insights into neural mechanisms governing responses to different colors across various frequency and time bands. Specifically, our results highlight distinctions in the perception of RGB colors within the delta, theta, and beta frequency bands, notably in occipital and frontal regions; see Figs. 3, 4 and 5. These findings offer valuable insights from both perceptual and cognitive standpoints, shedding light on the intricate interplay between color and neural processes.

In our investigation of the time-dependent effects of RGB color perception on brain activity using EEG signals, we notified that the beta-band event-related oscillation of occipital (O1, OZ, and O2) electrodes showed a significant latency shift of approximately 100 ms for green-colored stimuli compared to blue and red (Fig. 3). This novel finding warrants further exploration in future studies, as no prior research has reported a similar latency shift in RGB event-related oscillations. One possible explanation could be the differential processing of colors in distinct brain regions or pathways, which could account for this timing disparity. Additionally, the literature supports the notion that the choice of color may influence EEG decoding accuracy in color-perception tasks, as seen in a study using red and green-colored stimuli where it is reported that the beta-band provided the highest EEG decoding accuracy of color-perception tasks vs. mental-rotation tasks^77^ while using red and green-colored stimuli. Moreover, the observation of faster beta frequency responses to red and blue colors compared to green may be indicative of differences in alertness. Previous research by John and colleagues has demonstrated a link between increased beta band activity in parieto-occipital regions and heightened alertness, as evidenced by faster responses to visual stimuli tasks.Behavioral studies have also revealed noteworthy findings in this context^56^. For instance, red stimuli have been shown to possess an attentional advantage, as discussed in a comprehensive review by Folk^78^. Similarly, the literature suggests that exposure to blue light can enhance subjective alertness and performance on attention-based tasks, as outlined in the review by Chellappa et al.^79^. Hence, our findings may suggest that the relatively slower occipital beta band oscillations associated with green stimuli, in comparison to red and blue, could imply a lesser impact on alertness and attention. This aligns with the notion that red and blue may be more attention-capturing and alertness-enhancing colors, emphasizing the potential significance of our findings for understanding the interplay between color perception and cognitive processes.

Building upon previous studies on human and non-human primates and the available evidence showing noticeable effects on color perceptions in gamma-band rhythmic activity, our results showed no significant difference in gamma-band oscillations of EEG signals in RGB space (Supplementary 1). We could explain this result with the effect of different color spaces and luminance on the input of visual color information processing associated cortices, which in our case produced no evident induced gamma activity of RGB colors in EEG signals.

In addition, another significant result of our study showed a substantial difference in the power of theta-band oscillation in the occipital electrodes for blue-colored stimuli (Fig. 4). This result is interpretable from a psychological perspective and the effect of colors on emotional states. There are reports of considerable theta power changes in response to colored stimuli. Increased theta power is reported upon negative emotions compared with positive emotions^80^. Moreover, the blue color is suggested to have a less arousing effect due to an increment of theta power at the midline parietal regions relative to red stimuli^16^. Theta and gamma activity are observed in meditation studies without operational tasks in the posterior visual regions, which is correlated with attentional concentration^81,82^. Subsequently, our result showed that in contrast to red and green colors, blue-colored stimulus produces a remarkably negative power value relative to baseline activity during the late period post-stimulus onset, which holds for a relatively long time. Thus, our observation may stem from the emotional effect of blue-colored stimuli, producing a calming state of mind for the subjects. Furthermore, in temporal dimensional notions, it is more likely that arousal may influence the late (200–1000 ms) components^83^. Therefore, this notable late theta power decline probably demonstrates less arousal effect of blue color.

We also investigated the phase coherency values of the different frequency bands with ITPC analysis. Interestingly, the result significantly affected the delta-band frequency in prefrontal areas. During particular periods, the ITPC analysis showed that in contrast to other colors, only green-colored stimulus produces positive delta-band ITPC values (Fig. 5B and C). Nevertheless, phase coherence represents regulating the brain’s response to the temporal configuration of task-related information, which optimizes the data processing^84–87^. Some authors suggest that the higher degree of phase consistency presentation across trials may imply an association of attention^88,89^ and visual perception^90^. On the other hand, several studies suggest the involvement of delta oscillations in many cognitive processes^91^. Moreover, motivational processes, higher emotional involvement, and cognitive processes related to detecting salient stimuli in the environment^92–94^ are affected by the functional delta oscillations with implications in the synchronization of brain activity. In other studies^95,96^, delta has also been related to behavioral inhibition. Furthermore, it is proposed that low-frequency oscillations are associated with emotional processes^92^. Therefore, based on the observation of significant differences in the phase coherency value of colors, we believe that there may exist an underlying inhibition mechanism in different color stimulation’s delta-band activity, which could produce a variety of emotional states. Additionally, the color information could be transmitted through phase synchronization of different areas associated with these processes, and we suggest further investigation of these results for psychological purposes. Furthermore, we also observed that during late periods of prefrontal theta phase consistency, there was a decrease in ITPC values of green color (Fig. 5D and E), which needs further investigation in future studies.

## Conclusions

In summary, our study is about color perception, focusing on the RGB color space and employing a data-driven approach. Through the analysis of EEG signals, we aimed to unravel the nuances of how the human brain processes and responds to colors - red, green, and blue. Our findings have unveiled several noteworthy insights into the cognitive mechanisms underlying color perception. They collectively suggest that beta-band oscillations, theta-band power in the occipito-parietal region, and prefrontal phase consistency within delta and theta-bands may offer valuable indicators for tracking RGB color information and understanding the neural processes associated with color perception.

There are several potential research directions for the future works. Connectivity analyses such as phase-based connectivity, power-based connectivity, Granger causality, mutual information, cross-frequency coupling, and graph theory could provide further insights into the complex interplay between colors and brain activity. Expanding the scope of our study to encompass combinations of RGB colors could deepen our understanding of color perception in a broader context.

Acknowledging the limitations of our current work, we conducted a pilot study with a limited selection of RGB colors and a relatively small sample size. Extending our investigation to include a wider spectrum of colors and increasing the sample size could enhance the robustness and generalizability of our findings. Moreover, the controlled experimental environment, while necessary for scientific precision, may not fully represent real-world conditions, and future studies in more naturalistic settings could provide additional perspectives. Lastly, we acknowledge the gender imbalance in our participant pool due to the number of female participants (3 females) compared to the number of male participants (9 males). Although we show the separate results of theta power in occipital, and delta and theta phases in prefrontal for male and female subjects in Supplimentary 3, the matter is still open to further investigation in future works.

In conclusion, our study represents a significant step toward unraveling the enigmatic relationship between color perception and brain activity in the RGB color space. These findings not only contribute to our understanding of human cognition but also hold the promise of practical applications in design, marketing, and other fields where color plays a vital role.

### Supplementary Information

Supplementary Figures.

## Data Availability

The Data and code may be provided to interested researchers upon reasonable request to the corresponding author.
