# Supplementary material for: Brain activity characteristics of RGB stimulus: an EEG study
Source: Sci Rep. 2023 Nov 3;13:18988. doi: 10.1038/s41598-023-46450-z (PMC10624840; doi:10.1038/s41598-023-46450-z)
Supplement: Supplementary file 1 — Supplementary Figures. [file 41598_2023_46450_MOESM1_ESM.pdf]

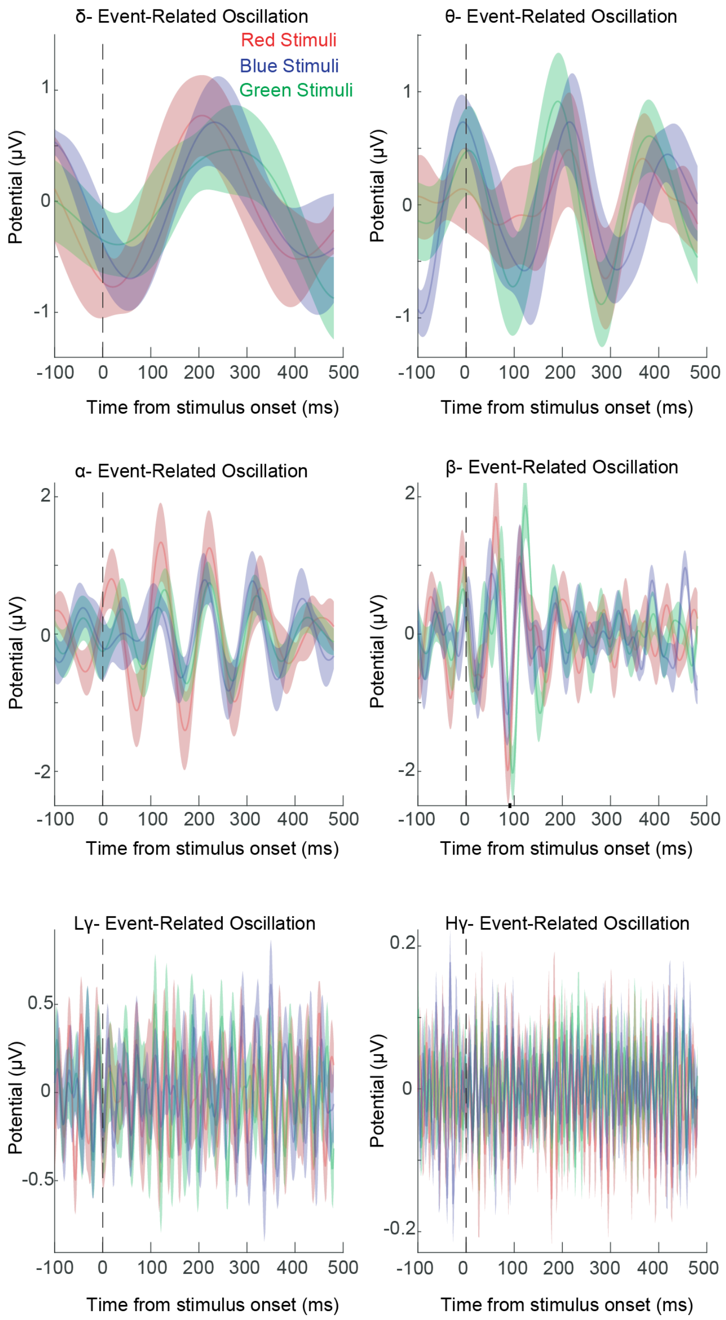

Supplementary 1. Event-related oscillation in occipital (O1, OZ, and O2) electrodes for all band frequencies (delta, theta, alpha, beta, low gamma, high gamma) | . Each line shows ERP values over time. Dash lines indicate the time of stimuli onset with Error bars showing 95% confidence intervals, calculated across participants (n = 12). As depicted, there is only a significant difference in ERO of beta-band activity between colors, approximately at 100 ms post-stimulus onset. In contrast, other frequency bands showed no significant effect. In the beta band, the Horizontal black lines show the clusters of significant differences between RGBs ERO in the time window of 88 to 98 ms (a within-subject Friedman Test, permutation test with n=1000 permutations, participant = 12, cluster-forming threshold $p < 0.01$, corrected significance threshold $p < 0.05$).

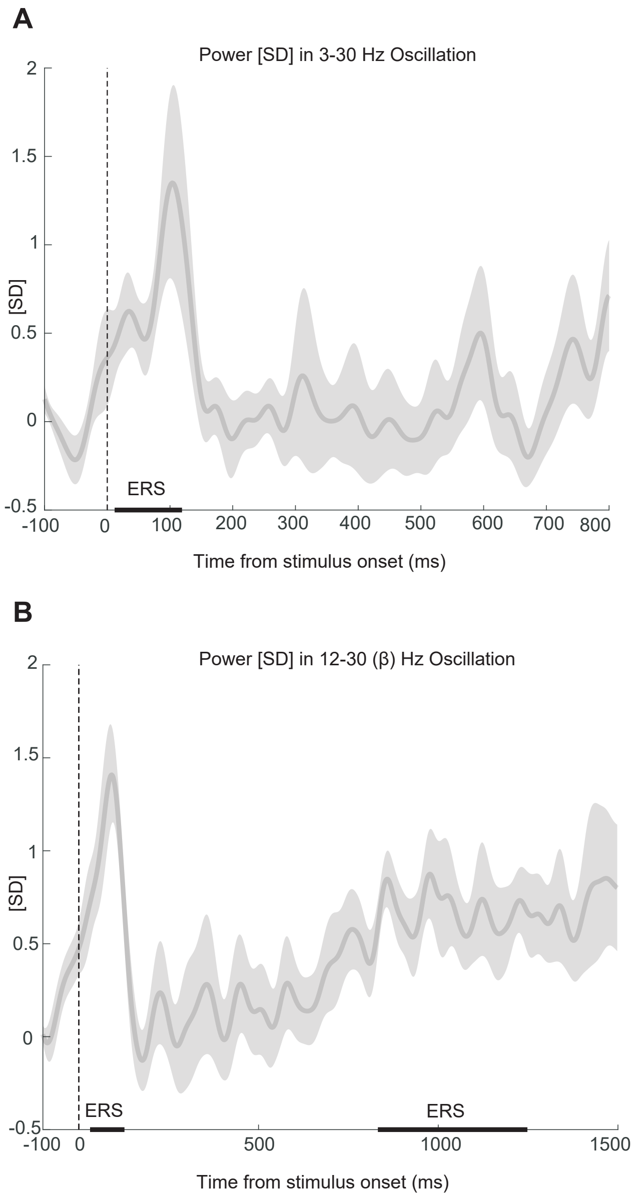

Supplementary 2. Event-related synchronization (ERS) in the beta-band (12-30 Hz) and 3-30 Hz in occipital (O1, Oz, and O2) electrodes — A) ERS for averaged over all color conditions for 3-30 Hz oscillation. Dash lines indicate the time of stimuli onset. Error shadings show 95% confidence intervals, calculated across participants (n = 12). The Horizontal black line shows the clusters of significant periods in which we can see ERS increase in the time window of 11-120 ms (Wilcoxon tests, 1000 permutations, cluster-forming threshold Z >1.96, the corrected threshold p <0.05). B) Similar to A, for the beta band (12-30 Hz). The Horizontal black line shows significant occurring ERS in the time window of 30-127 ms and 830-1240 ms (Wilcoxon tests, 1000 permutations, cluster-forming threshold Z >2.56, the corrected threshold p <0.05)

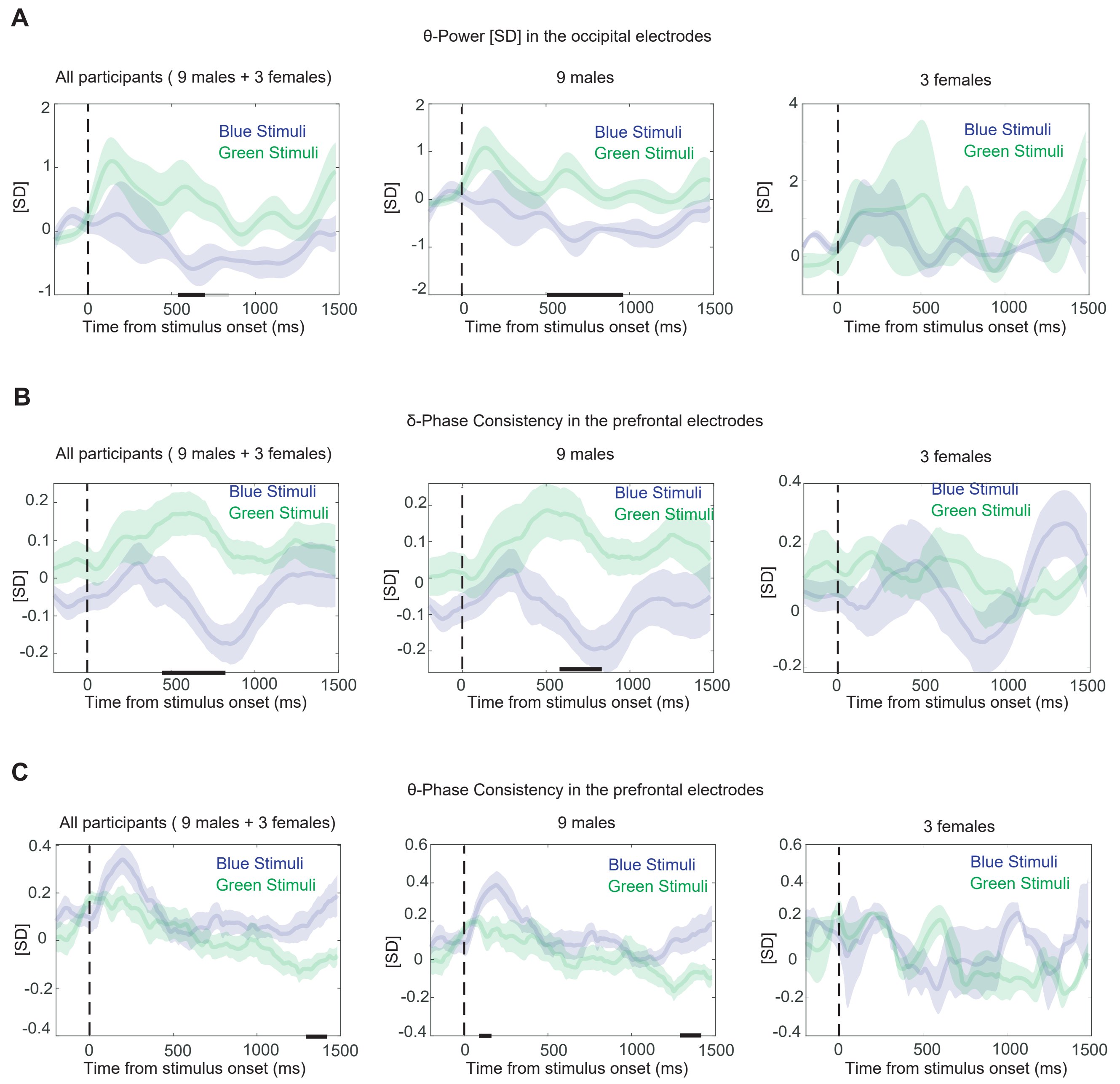

Supplementary 3. **Separate results of theta power in occipital (A), and delta in prefrontal (B) and theta in prefrontal (C) for male and female subjects**.
